# Conflicting Role of *Mycobacterium* Species in Multiple Sclerosis

**DOI:** 10.3389/fneur.2017.00216

**Published:** 2017-05-19

**Authors:** Davide Cossu, Kazumasa Yokoyama, Nobutaka Hattori

**Affiliations:** ^1^Juntendo University, Tokyo, Japan

**Keywords:** multiple sclerosis, heat shock protein, molecular mimicry, *Mycobacterium tuberculosis* complex, *Mycobacterium avium* subsp. *paratuberculosis*, mycobacteria-associated molecular pattern-recognition signals

## Abstract

*Mycobacterium* is a genus of aerobic and acid-fast bacteria, which include several pathogenic organisms that cause serious diseases in mammals. Previous studies have associated the immune response against mycobacteria with multiple sclerosis (MS), a chronic demyelinating disease of the central nervous system with unknown etiology. The role of mycobacteria in the pathological process has been controversial and often conflicting. We provide a detailed review of the mycobacteria that have been linked to MS over the last three decades, with a focus on *Mycobacterium bovis* bacille Calmette–Guérin vaccine for human and oral exposure to *Mycobacterium avium* subsp. *paratuberculosis*. We will also discuss the exposure and genetic susceptibility to mycobacterial infection, the protective role of vaccination, as well as the possible mechanisms involved in initiating or worsening MS symptoms, with particular emphasis on the molecular mimicry between mycobacterial and human proteins. Finally, we will introduce topics such as heat shock proteins and recognition by innate immunity, and toll-like receptor signaling-mediated responses to *Mycobacterium* exposure.

## Introduction

Multiple sclerosis (MS) is a chronic, inflammatory demyelinating disorder of the central nervous system (CNS) characterized clinically by a wide variety of neurological symptoms (1).

Even though rigorous research has been performed in the MS field, its etiology as well the exact pathogenic mechanisms remains poorly understood. Nevertheless, it is believed to be a multifactorial disease caused by autoimmune processes (1). While the genetic susceptibility to MS is strongly correlated with the human leukocyte antigen (HLA) class II, the low concordance rate between monozygotic twins (from 5 to 25%) suggest an equally important role for the environment in inducing symptoms (1).

Clinical evidence suggests that an autoimmune response directed against myelin, possibly stimulated by an infectious agent, contributes to break tolerance (2). Literature related to risk factors for MS has grown significantly in recent years, and several infectious pathogens, including herpes viruses such as the *Epstein–Barr* virus (EBV) (2, 3), endogenous retroviruses (3), as well as bacteria like *Helicobacter pylori, Chlamydia pneumoniae*, and different mycobacteria, have been identified, even if their roles in the pathology remain controversial (2).

## Mycobacteria and Toll-Like Receptors (TLRs) in MS

**Figure 1:**
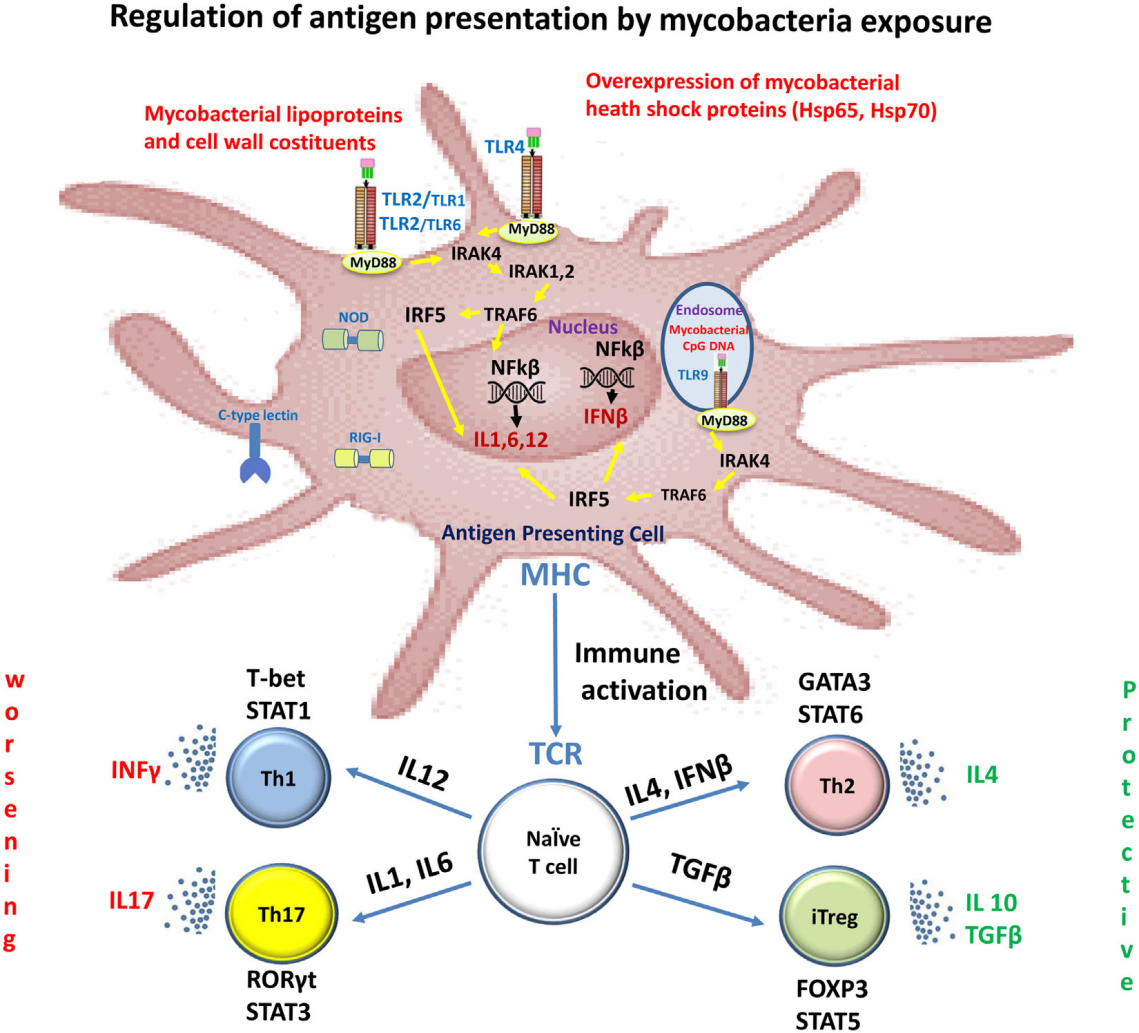
**Immune recognition of mycobacterial components by toll-like receptors (TLRs) in multiple sclerosis (MS)**. Mycobacterial components are potent activators of the innate immune system via TLRs. Stimulation of the host immune response with TLR2 and TLR4 induces the production of IL1, IL6, and IL12. These cytokines induce the differentiation of naïve CD4+ T cells into Th1 and Th17 cells. IFNγ and IL-17, produced by Th1 and Th17 cells, respectively, facilitate leukocyte transmigration across the blood–brain barrier, thereby contributing to tissue damage and neuronal dysfunction. Dendritic cells present in cerebrospinal fluid and lesions of MS patients are activated with TLR9 agonists and they promote Th1 and Th17 responses. TLR2 signaling also affects both expansion and function of Tregs, a T-cell subset that plays a crucial role in the control of autoimmune diseases such as MS.

Innate immunity plays an important role in the initiation and progression of MS, as well as in host defense against mycobacteria infection and their immunogenic components (Figure 1) (4).

The TLR2, TLR4, and TLR9 are the main members of the TLR family that recognize mycobacterial components (5); the innate immune system cells contributing to MS pathology express all the TLRs. Several studies have shown that these receptors, especially the TLR2, TLR4, and TLR9, have a fundamental role in MS pathology (6). Indeed, their expression levels are increased in MS as well as experimental autoimmune encephalitis (EAE), an animal model of MS. For example, TLR2 ligands such as peptidoglycan, a major component of mycobacterial cell walls, have been identified in CNS endothelial cells, cerebrospinal fluid (CSF), microglia, astrocytes, oligodendrocytes, and on infiltrating cells in patients with MS (7, 8). The induction of several models of EAE requires complete Freund’s adjuvant containing *Mycobacterium* and pathogen-associated molecular patterns that activate innate immune responses through TLRs such as TLR2, TLR4, and TLR9 (9).

## Mycobacterial Heat Shock Proteins (HSPs) and MS

Heat shock proteins are a family of molecular chaperones that play a role in innate immunity as they can interact with TLRs, but they are also involved in acquired immunity processes, enhancing antigen presentation and the activation of lymphocytes and macrophages (10).

Heat shock protein families are highly conserved throughout evolution, and immunogenic mycobacterial proteins share sequential and conformational elements with several human host proteins. Mycobacterial HSP expression has been shown to increase under conditions of stress, and many antigenic HSPs are common targets of humoral and cell-mediated immune responses in humans, resulting in immunological cross-recognition between human and microbial homologs. This process, also called molecular mimicry, has been hypothesized to initiate and exacerbate the autoimmune response mounted against antigens by pathogens with sequence or structural similarities with homologous self-proteins (11).

Animal studies have shown that a booster dose with a DNA vaccine containing the mycobacterial HSP65 gene after bacille Calmette–Guérin (BCG) priming can promote protection against EAE (12).

Regarding their potential role in MS, various studies have shown a more frequent lymphocyte proliferative response against recombinant proteins, HSP65 and HSP70, derived from *Mycobacterium tuberculosis* and *Mycobacterium leprae* in MS, compared to that against specimens isolated from patients with other neurological diseases or healthy control subjects (13–15).

Another study also demonstrated an increased level of circulating *Mycobacterium avium* subsp. *paratuberculosis* (MAP) HSP70 antibodies in Sardinian patients with MS (16).

## Genetics of MS and Mycobacteria

At present, there are no genome-wide association studies testing genetic susceptibility of patients with MS to mycobacteria. Case–control studies of individuals with increased susceptibility to BCG and other atypical mycobacterial infections have identified different host genetic factors, including polymorphisms in the major histocompatibility complex, TLR, vitamin D receptor genes, genes encoding IFN-gamma signaling components, and *SLC11A1* (17). Majority of these genes have a pivotal role in determining susceptibility to mycobacteria infection and are related to the pathogenesis of autoimmune diseases, including MS (18).

In addition, recent studies have reported a higher frequency of anti-MAP antibodies in Sardinian patients with MS with predisposed DRB1-DQB1 HLA (*04:05-*03:01, *03:01-*02:01) (19), and a lower frequency of anti-MAP antibodies in patients with MS with protective DRB1-DQB1 HLA (*15:02-*06:01, *16:01-*05:02, and *14:01-4-*05:03) (20).

## MAP and MS

*Mycobacterium avium* subsp. *paratuberculosis*, an intracellular pathogen belonging to the *Mycobacterium avium* complex, is the causative agent of paratuberculosis or Johne’s disease (2) in ruminants. Despite the controversy regarding the role of MAP as a zoonotic agent in human diseases, several studies have demonstrated a link between MAP and other autoimmune diseases, such as MS (2).

The association between MAP and MS was established for the first time in Sardinia, an Italian Mediterranean island, to which MAP is endemic (21). Furthermore, a recent seroprevalence study confirmed the association between MAP and MS in Japan (22). Geographically, people who live farther from the equator have a higher risk of developing MS than people living in hot areas near the equator. Sardinia is a notable exception, as it reports one of the highest prevalence rates of MS in the world (2) and near the equator. Japan not only shows a low prevalence of MS compared to Caucasian countries but also reports a low prevalence of MAP-infected individuals (23), given the strict quarantine system. Considering the dearth of epidemiological studies of MAP, the pattern of MS prevalence does not seem to parallel the distribution of MAP in the world.

As humans are not the natural host of MAP, it was hypothesized that MAP exposure from cattle to humans primarily occurs via the fecal–oral route. Due to its thermal resistance, MAP can survive the pasteurization process, thereby rendering contaminated milk and dairy products as vehicles for the transmission of its antigenic fractions (22, 23).

The first evidence supporting this hypothesis was the detection of MAP DNA in the peripheral blood mononuclear cells (PBMCs) of 15.5% of the 436 patients with MS, and in 2.3% of the 264 healthy controls by PCR amplification of the highly specific *IS900* sequences (24).

Further, the possible involvement of MAP in the etiology and pathogenesis of MS was supported by the discovery of several MAP proteins and antigenic peptides capable of inducing a stronger humoral and cell-mediated response in the PBMCs of patients with MS than in those of control subjects (16, 22).

The humoral response against several homologous epitopes of EBV, MAP, and human proteins has also been explored (25–27). Antibodies against these antigens were found in the CSF and serum of matched MS samples (28). The fact that peptides from different pathogens can be cross-recognized by antibodies targeting self-epitopes supports the hypothesis that more than one pathogen is implicated in MS etiology, and in the case of EBV and MAP peptide homology, they might trigger autoimmunity through a common target like myelin basic protein (MBP).

Special attention has been given to MAP_2694_295-303_, an immunodominant B-cell epitope belonging to a MAP-specific protein, which is located within the high homology region with the T-cell receptor gamma-chain protein (22). Gamma-delta T-cells are a family of cells that take part in both innate and adaptive immunity and play a pathogenic role in CNS inflammation and autoimmunity (22). Antibodies against MAP_2694_295-303_ were detected in the sera of patients with MS in Sardinia and Japan (19, 22).

## BCG Vaccine and MS

*Mycobacterium bovis* belongs to the *M. tuberculosis* complex and is the causative agent of tuberculosis in cattle, but it can sometimes cause tuberculosis in other mammals, including humans.

Bacille Calmette–Guérin is a live attenuated strain of *M. bovis*, and it is mainly used as a vaccine against tuberculosis. Systemic infection with BCG can suppress autoimmune responses in EAE in mice by redirecting trafficking of activated CNS antigen-specific CD4+ T cells to local inflammatory sites induced by BCG infection (29). In an EAE model induced by MOG_35-55_ peptides in mice, intracerebral infection of live BCG suppressed the development of CNS autoimmune encephalomyelitis (30). BCG-infected mice with *M. bovis* had fewer infiltrating mononuclear cells in the spinal cord, but also fewer myelin oligodendrocyte glycoprotein (MOG)-specific IFN-γ+, CD4+, and IL-17+CD4+ T cells following EAE induction (30). Similar results were obtained with the *in vivo* administration of *M. tuberculosis* prior to immunization with MBP, leading to suppression of the immune response and reduction in the severity of EAE through the production of TGF-beta by gamma-delta T cells (31).

Clinical trials using the BCG vaccine as an adjuvant therapy for patients with MS have shown beneficial effects of vaccination. A crossover trial revealed that a single BCG vaccination decreased magnetic resonance imaging-based disease activity in a cohort of 12 relapsing remitting patients with MS (32).

In a double-blind placebo-controlled trial including vaccinated subjects and placebo controls, BCG was associated with significantly reduced development of gadolinium-enhancing lesions in patients with clinically isolated syndrome (CIS) for a 6-month period before starting the immunomodulation therapy (33). CIS describes a first clinical episode of neurological symptoms that lasts at least 24 h, with features suggestive of MS. In the same study, BCG vaccination appears to have a long-term effect in patients with CIS; compared to placebo-treated patients, BCG-treated people showed fewer T1-hypointense lesions and lower cumulative number of relapses after 18 months, in addition to lower risk of conversion to clinically definite MS over a period 5 years. However, there are no data demonstrating its effect on patients with progressive MS.

Few studies have investigated the humoral response elicited by BCG antigens. A seroprevalence study based on the antibody detection against the encephalitogenic MOG_35-55_ epitope and BCG-derived homologous peptides showed the absence of antibody-recognition against BCG peptides in 40 Sardinian patients with MS (34).

## Conclusion

Overall, our findings demonstrated a relationship between mycobacteria and MS. Cell-mediated immunity plays a critical role in protection against mycobacteria as they are intracellular pathogens, and even if the exact mechanisms underlying the effects of BCG on neuroinflammation are unclear, a protective role of BCG vaccination on MS progression is generally accepted. Further research is needed for potential therapeutic use of the BCG vaccine in patients at risk of developing MS.

*Mycobacterium avium* subsp. *paratuberculosis* seems to have a causal role in the MS pathology according to the molecular mimicry theory in some genetically predisposed individuals, and clinical trials of antimycobacterial therapy targeting MAP are currently under way (35). The role of mycobacteria in the initiation and progression of MS could be a population-specific phenomenon, highly dependent on different genetic and non-genetic factors. Immune modulation as a strategy to combat mycobacterial infection remains underexplored.

## Author Contributions

DC performed the experiments related to several articles included in this mini review and wrote the manuscript; KY wrote the manuscript and supported in the critical reading; NH wrote the manuscript and supported in the critical reading.

## Conflict of Interest Statement

The authors declare that the research was conducted in the absence of any commercial or financial relationships that could be construed as a potential conflict of interest.
